# Monkeys perform as well as apes and humans in a size discrimination task

**DOI:** 10.1007/s10071-013-0616-0

**Published:** 2013-02-27

**Authors:** Vanessa Schmitt, Iris Kröger, Dietmar Zinner, Josep Call, Julia Fischer

**Affiliations:** 1 Cognitive Ethology Laboratory, German Primate Center, Kellnerweg 4, 37077 Göttingen, Germany; 2 Courant Research Centre “Evolution of Social Behaviour”, University of Göttingen, Kellnerweg 6, 37077 Göttingen, Germany; 3 Max Plank Institute for Evolutionary Anthropology, Deutscher Platz 6, 04103 Leipzig, Germany

**Keywords:** Great apes, Baboons, Macaques, Humans, Cognition, Brain size

## Abstract

Whether the cognitive competences of monkeys and apes are rather similar or whether the larger-brained apes outperform monkeys in cognitive experiments is a highly debated topic. Direct comparative analyses are therefore essential to examine similarities and differences among species. We here compared six primate species, including humans, chimpanzees, bonobos, gorillas (great apes), olive baboons, and long-tailed macaques (Old World monkeys) in a task on fine-grained size discrimination. Except for gorillas, subjects of all taxa (i.e. humans, apes, and monkeys) were able to discriminate three-dimensional cubes with a volume difference of only 10 % (i.e. cubes of 50 and 48 mm side length) and performed only slightly worse when the cubes were presented successively. The minimal size discriminated declined further with increasing time delay between presentations of the cubes, highlighting the difficulty to memorize exact size differences. The results suggest that differences in brain size, as a proxy for general cognitive abilities, did not account for variation in performance, but that differential socio-ecological pressures may better explain species differences. Our study highlights the fact that differences in cognitive abilities do not always map neatly onto phylogenetic relationships and that in a number of cognitive experiments monkeys do not fare significantly worse than apes, casting doubt on the assumption that larger brains per se confer an advantage in such kinds of tests.

**Electronic supplementary material:**

The online version of this article (doi:10.1007/s10071-013-0616-0) contains supplementary material, which is available to authorized users.

## Introduction

To understand the evolution of differing cognitive traits, comparative analyses across a wide range of taxa are needed (MacLean et al. 2011; Menzel and Fischer 2011; Nunn 2011). With regard to the evolution of primate intelligence, comparisons between monkeys and great apes are particularly informative (Amici et al. 2008, 2010, 2012; Schmitt et al. 2012). These phylogenetic groups have long been considered to exhibit large differences in cognitive competences, not least due to differences in their brain sizes (e.g. Byrne 2000; Deaner et al. 2007, but see Tomasello and Call 1997). Great apes have relatively larger brains than monkeys (Jerison 1973), giving rise to the notion that apes outperform their primate relatives in a wide range of cognitive domains, such as causal understanding or tool use (Deaner et al. 2006). Recent studies, which revealed only slight differences between apes and monkeys in a variety of cognitive tests, however, challenge this assumption (Amici et al. 2010; Schmitt et al. 2012).

In particular, physico-cognitive abilities such as discriminating between quantities or remembering the location of hidden food seem to be shared between monkeys and apes (Amici et al. 2010; Schmitt et al. 2012). In contrast to the discrimination of different quantities, which has been extensively tested in a large number of studies and species (see Nieder 2005 for a review; Schmitt and Fischer 2011), the discrimination of objects of different size has rarely been examined. Yet, animals are confronted with items that vary in size throughout their lives, such as foods, conspecifics or predators, and the ability to discriminate items on the basis of their size is assumed to be highly advantageous. Sexual selection theory for instance predicts that females should mate selectively with high-quality males and choose their mates according to signals that reliably indicate male quality (Kappeler and van Schaik 2004). One predictor of male quality is body size, because it shows that (1) the male was able to accumulate sufficient nutrients and energy to grow to its respective size and (2) larger-bodied males may have a higher resource holding potential and may be more successful competitors (Andersson 1994; Trivers 1972). Choosing the larger male consequently may increase a female’s reproductive success and her fitness. A study by Caillaud et al. (2008) in gorillas (*Gorilla gorilla gorilla*) indeed showed that the size of males’ sagittal crest, which indicates its strength and health status, positively correlated with the number of females belonging to a male.

The importance of discriminating different sized objects may differ among species depending on factors such as ecology or mating system. Nonetheless, behavioral studies on visual size discrimination comparing the performance of different species and accounting for the possible influences of ecological factors are rare and only few psychophysical studies investigated the actual abilities of animals in this regard (see Cloarec 1986 studying insects; Mishkin and Hall 1955 for a brain lesion study in monkeys; Simon et al. 2006 for a study on echolocation-based size discrimination in bats; see also the growing interest in studies on visual illusions, Suganuma et al. 2007; Tudusciuc and Nieder 2010). Nevertheless, differently sized objects have been used in a number of cognitive tests, such as in studies of relational learning (e.g. Hauf 2008; Sarris et al. 2001). For instance, Kennedy and colleagues (Flemming and Kennedy 2011; Kennedy and Fragaszy 2008) used objects of different size to test whether capuchin monkeys and chimpanzees are able to match a demonstrator’s action to find hidden food. Whereas the chimpanzees performed well, only one capuchin mastered the task, supposedly showing species differences in analogical reasoning. However, as the size differences between the objects used were rather small, the results may be influenced by perceptual rather than cognitive differences. In other words, the capuchin monkeys may have been unable to discriminate between the differently sized objects, hindering them to understand the actual task (see also Bshary et al. 2011 for a discussion on incorporating perceptual characteristics in cognitive studies).

But not only environmental aspects or brain size may influence species performances in discrimination experiments. Similar performances may also be due to phylogenetic relatedness, as a specific competence may be inherited by all species through common descent (MacLean et al. 2011; Nunn 2011). Identifying similarities and differences in the cognitive abilities of closely related species is therefore a prerequisite to achieve a better understanding of possible selective pressures on specific abilities. To assess size discrimination from a comparative perspective, we tested six closely related primate species including humans, apes, and monkeys that differed in brain size, mating system, and ecological factors such as diet. Specifically, we included human subjects, three other great ape species (chimpanzees *Pan troglodytes*, bonobos *P. paniscus*, gorillas *Gorilla gorilla*), and two Old World monkey species (olive baboons *Papio anubis*, long-tailed macaques *Macaca fascicularis*) in our study. The subjects were tested in two-choice tests in which they were rewarded for choosing the larger of two cubes, which were presented simultaneously. Because under natural conditions, objects are not always fully visible at the same time, we included a second condition in which the two cubes were not shown simultaneously to the subjects but in succession (increasing the time interval from 5 to 20 to 60 s).

If phylogenetic relatedness or brain size, which is often considered as a proxy for general intelligence (Reader et al. 2011), had an influence on the performance of the species, then monkeys should perform worse than apes, which in turn should perform worse than humans having the largest brains. As other studies, however, indicated that differential socio-ecological factors can also influence the performance in cognitive experiments (Amici et al. 2008), we expected to find differences within the phylogenetic groups, which may be better explained by socio-ecological factors rather than by phylogenetic relatedness.

## Experiment 1: small size discrimination

In this experiment, we tested the fine-grained size discrimination abilities of humans, other apes, and monkeys. In the first part of the experiment, the subjects had to discriminate between a pair of three-dimensional cubes that differed in volume when these were presented simultaneously. In the second part of the experiment, the cubes were only shown in succession, that is, one after the other, with three different time delays between presentations.

### Methods

#### Subjects

##### Human participants

We tested eight adult humans—4 men and 4 women aged 26–57 years (mean = 35.3 years). All subjects participated voluntarily.

##### Apes

Five chimpanzees, five bonobos, and eight gorillas participated in this study—6 males and 12 females with an age of 7–28 years (Online Resource 1).

With the exception of 3 gorillas (Bianka, Hakuna, Lena) who lived at the Nürnberg Zoo, Germany, the apes were housed at the Wolfgang Koehler Primate Research Center in Leipzig Zoo, Germany. The apes lived in social groups and had access to indoor and outdoor enclosures. Subjects were individually tested in a familiar testing room (chimpanzees and gorillas) or in their sleeping cages (gorillas and bonobos). Water was always available ad libitum, and subjects were not food deprived for testing. All apes except those housed at the Nürnberg Zoo were familiar with experimental testing situations.

##### Monkeys

Nine olive baboons and eight long-tailed macaques—6 males and 11 females with an age of 2–11 years—participated in this study (Online Resource 1). One baboon (Nase) dropped out of the study because she was transferred to another facility. The long-tailed macaques lived in a social group of 28 animals. The olive baboons lived in a social group of 11 animals. The monkeys were housed at the German Primate Center in Göttingen and had access to indoor (baboons: 17 m², macaques: 40 m²) and outdoor areas (baboons: 81 m², macaques: 141 m²).

Subjects were individually tested in their familiar indoor enclosure. Water was always available ad libitum, and subjects were not food deprived for testing. None of the baboons had experience in cognitive experiments, whereas the macaques had already participated in previous studies (Schmitt and Fischer 2011; Schmitt et al. 2012).

#### Materials

A set of 9 equilateral cubes of different volumes (Table 1) were used. The cubes were built of pink cardboard and covered with transparent adhesive plastic film. One side of the cube was open so that the cubes could be placed over a food reward (grape or peanut). The cube with an edge length of 50 mm was set to represent 100 % (Table 1). This cube was then used as a reference to adjust the size of the other cubes.

**Table 1:** Cube set used to test the size discrimination abilities of the humans, apes and monkeys

Size	140 %	130 %	120 %	110 %	100 %	95 %	90 %	80 %	70 %
Edge length (mm)	58	56	54	52	50	49	48	46	44
Volume (cm^3^)	195	176	157	141	125	118	111	97.3	85.2

For the nonhuman subjects, a sliding table was used to place the cubes in front of the subjects. To do so, a sliding board was attached to a table so that the board could be moved horizontally. The table was attached with an iron mount in front of a plastic pane. Two cubes were placed on the right and left side of the sliding board. Two holes (apes: diameter 35 mm, distance from center to center 560 mm; monkeys: diameter 15 mm, distance 300 mm) in the plastic panel allowed the subjects to point with their fingers at the cubes. For the human subjects, the cubes were placed on a normal table and the subjects pointed with their fingers at the designated cube. Additionally, two blue plastic cups (height 75 mm, diameter 90 mm) were used to cover the pink cubes in the successive conditions. In addition, an occluder could be set up in front of the panel so that the subject was not able to watch the baiting of the cubes. All trials with the nonhuman subjects were videotaped.

#### Test design

##### *Nonhuman primates*

Simultaneous presentation

Each subject was first tested in the simultaneous condition. Here, every trial consisted of the following elements: The sliding table was removed from the panel, and the occluder was positioned to hide the setup. The experimenter showed a food reward (grape or peanut) to the subject and then placed the reward on the sliding table where the subject was no longer able to see it. Then, the experimenter showed the two cubes with the open side toward the subject so that it could see the cubes where empty. Next, the experimenter covered the reward with the larger cube and placed one cube to the right and the other to the left side of the sliding table (pseudorandomly, with the restriction that the reward should not appear on the same side for more than two consecutive trials but equally often left and right). The experimenter removed the occluder and pushed the table to the panel. The subject was allowed to choose one of the cubes by pointing at it through the holes in the panel. If the subject chose the bigger cube, it received the reward; otherwise, it received nothing, but was shown the place of the reward.

Each session consisted of 12 trials with the larger cube being equally often on the left and the right side of the table. A session was scored as “passed”, if the subject chose the larger cube in more than 10 trials, that is, 11 or 12 times correct. Each subject received a maximum of 12 sessions per volume difference.

Every subject started with a volume difference of 30 % (Table 2). If the subject reached criterion twice with this volume difference (i.e. passed two sessions), the condition was scored as “passed” and the volume difference was decreased. If the subjects did not reach criterion within the 12 sessions, the condition was scored as “failed” and the subject was not tested further. The volume difference was progressively decreased until the subject either failed the condition or reached the 5 % volume difference condition. Afterward the subject was tested using successive presentations of the stimuli.

**Table 2:** Cube combination for the subjects (only the baboons were tested with the 40–100 % volume differences)

Difference	100 %	80 %	60 %	40 %	30 %	20 %	10 %	5 %
Cube combination (edge length in mm)	58 44	56 44	54 44	52 44	50 44	50 46	50 48	50 49

All of the baboons failed in the start condition of 30 % size difference. As none of the study individuals had prior experience with any experimental testing, we increased the size difference to 100 % (Table 2). The subjects who passed this condition continued with 80, 60 %, and so on until they failed. The rest of the procedure was the same as for the other species.

Successive presentation

The procedure was the same as in the simultaneous presentation, but additionally both cubes were covered with blue cups before the occluder was removed. Then, each cup was lifted one after the other for 3 s so that the subject could see the cube underneath. The time span between the hiding of the first cup and the lifting of the other was increased incrementally from 5 to 20 s and then 60 s. At the time of choice, both cubes were covered. Subjects were rewarded when they chose the cup under which the larger cube was hidden.

The successive presentations started with the volume difference the subject had passed last in the simultaneous presentations; that is, if a subject passed 10 % in the simultaneous presentation but failed the 5 % condition, it was first tested with 10 % volume difference and a time span of 5 s in the successive presentation. If the subject passed the condition (≥11/12 correct in two sessions), the time span was extended to 20 s and afterward to 60 s. If a subject did not pass one of the time intervals, the volume difference was increased (for example from 10 to 20 %), and the subject was tested with the respective time interval. If the subject now passed, the time interval was increased again until the 60 s interval was reached. If it failed, the volume difference was increased further (for example from 20 to 30 %) until the subject passed the time interval or failed in all conditions.

The baboons received a slightly different procedure to account for the different cube combinations in the simultaneous condition. As for the other species, the successive presentations started with the volume difference the baboon had passed in the simultaneous presentations. However, if a subject failed the 5 s delay with this size difference, we immediately increased the difference to 100 % (see Table 2). If the baboon passed, we then stepwise decreased the size difference until the subject failed in a size difference. We then increased the time delay to 20 and 60 s for the size difference the subject had passed last.

In these successive presentations, each subject of each species received a maximum of 12 sessions per condition, with 12 experimental trials and 2 motivational trials per session. In the motivational trials, the cubes were shown simultaneously to maintain a subject’s interest in the task.

##### *Humans*

As we were interested in the discrimination threshold of the human participants, we told the study participants that they would have to choose the larger of two cubes in the subsequent test conditions, but reported no further details of the testing procedure. As for the nonhuman primates, the experimenter put two cubes on a table in front of the subject in the simultaneous condition. After about 3 s, the participant indicated his/her choice by pointing at the cube. The experimenter then told the participant whether the choice was correct. The position of the larger cube was pseudo-randomized with the restriction that it should not appear on the same side for more than two consecutive trials, but equally often on the left and right. We tested the human participants with the 20, 10, and 5 % size difference between the cubes (see Table 2) in two 12-trial sessions per condition (we did not include the 30 % size difference as every participant was already able to discriminate the 20 % size difference between the cubes). To compare the results to the nonhuman subjects, a condition was scored as “passed” if a participant chose the larger cube 11 or 12 times correct in both sessions.

The successive condition was also the same as for the nonhuman subjects. The experimenter put the two cubes on the table and covered them with larger cubes behind an occluder. The occluder was removed, and the first cube was shown to the participant for about 3 s. The second cube was shown after a delay of 5, 20, or 60 s. Again, the position of the larger cube was pseudo-randomized with the restriction that it should not appear on the same side for more than two consecutive trials. The human participants were tested with each size difference (i.e. 20, 10, 5 %) in each time delay (i.e. 5, 20, 60 s) for two 12-trial sessions, resulting in a total of 18 sessions. In case a participant did not reach the criterion for the 20 % size difference within a given time delay, he or she was also tested with a 30 % difference between the cubes.

#### Data analyses

First, we compared the absolute number of subjects passing the initial simultaneous condition for each species. To be counted as “passed”, a subject had to choose the larger cube in more than 10 out of 12 trials in two sessions. Second, we assessed the minimal size difference each subject was able to discriminate, that is, the last condition in which it had passed two sessions. Because of the small sample size, we then conducted Kruskal–Wallis ANOVAs to test for differences between species and between phylogenetic groups (i.e. human, ape, monkey). In the successive condition, we compared the performances of the different species (and phylogenetic groups) in the different time delays (i.e. 5, 20, 60 s) using a repeated-measures ANOVA because of repeated testing of the same subjects. To compare the simultaneous and successive condition, we calculated the mean performances in both conditions and conducted a repeated-measures ANOVA to control for repeated testing. In case of significant results, we conducted a Holm–Sidak post hoc test. The alpha-level was set to 0.05.

### Results

#### Simultaneous discrimination

**Fig. 1:**
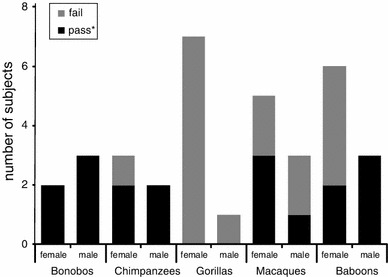
Number of subjects, which did and did not learn to choose the larger of two cubes in the simultaneous condition of Experiment 1. * ≥ 11/12 trials correct in two sessions

Figure 1 presents the number of subjects that passed (or failed) the simultaneous discrimination as a function of species. None of the gorillas learned to choose the larger of the two cubes, whereas all humans and 4–5 subjects of each other species passed this initial condition. In total, 13 females and 13 males chose the larger cube successfully.

**Fig. 2:**
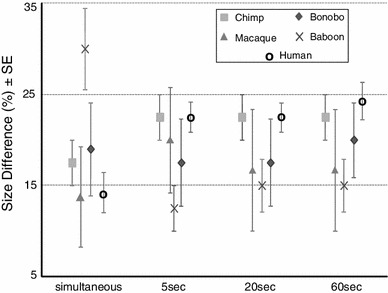
Mean (±SE) smallest size difference the different species were able to discriminate in the simultaneous and successive conditions of Experiment 1. The time period indicates the time passed between the presentations of the two cubes in the successive condition

Excluding gorillas, the subjects were able to discriminate between alternatives that differed on average 18.65 % ± 1.97 SE in size. Figure 2 shows that the baboons performed worst with a discriminated size difference of 30 % ± 4.47 (mean ± SE), whereas the macaques were able to discriminate the smallest size differences of 13.75 % ± 5.54 (mean ± SE). However, we found no significant differences between the performances of the successful species (Kruskal–Wallis ANOVA with species as between-subject factors: *H*_(4, *N*=26)_ = 7.45, *p* = .114) and no differences between apes, monkeys, and humans (Kruskal–Wallis ANOVA with phylogenetic group as between-subject factor: *H*_(2, *N*=26)_ = 2.19, *p* = .335), but rather relatively large individual differences within the different species as shown in Table 3. Three subjects were even able to discriminate reliably between cubes that differed only by 1 mm in edge length (5 % difference). Neither sex (two-way ANOVA with sex and species as between-subject factors: *F*_(1,16)_ = 1.91, *p* = .186) nor species x sex (*F*_(4,16)_ = 2.05, *p* = .136) had any influence in the lowest discrimination point reached by the subjects.

**Table 3:** Smallest size difference (in %) the subjects were able to discriminate in each condition and mean group performance for the different species (bold)

Condition	Simultaneous	5 s	20 s	60 s
**Chimpanzees**	**17**.**5**	**22**.**5**	**22**.**5**	**22**.**5**
Frodo	20	20	20	20
Patrick	20	20	20	20
Dorien	20	30	30	30
Natascha	*f*			
Fraukje	10	20	20	20
**Bonobos**	**19**	**17.5**	**17.5**	**20**
Joey	30	*f*		
Limbuko	10	10	10	20
Kuno	5	10	10	10
Ulindi	30	30	30	30
Yasa	20	20	20	20
**Gorillas**				
Gorgo	*f*			
Ndiki	*f*			
Bebe	*f*			
Viringika	*f*			
Bianka	*f*			
Hakuna	*f*			
Lena	*f*			
Ruby	*f*			
**Baboons**	**30**	**12**.**5**	**15**	**15**
Meister	40	10	20	20
Jago	40	10	10	10
Pünktchen	20	10	10	10
Tröpfchen	*f*			
Nase	30			
Schecki	*f*			
Brille	*f*			
Beinhaar	20	20	20	20
Tiger	*f*			
**Macaques**	**13**.**75**	**20**	**16**.**67**	**16**.**67**
Samson	*f*			
Pit	30	30	30	30
Lenny	*f*			
Sunny	*f*			
Maja	10	10	10	10
Sally	*f*			
Linda	*5*	30	*f*	
Sophie	10	10	10	10
**Humans**	**14**.**38**	**22**.**5**	**22**.**5**	**24**.**29**
C	10	20	20	20
Mm	5	20	20	20
Km	20	–	20	–
B	20	20	20	20
Mf	20	30	30	30
V	10	30	20	30
Kf	20	20	30	30
J	10	20	20	20

*f* = failed the condition

#### Successive discrimination

Except for one bonobo, all subjects that had learned to choose the larger cube in the simultaneous presentation were still able to discriminate between the stimuli when these were presented in succession. Comparing the performances of the subjects in the different time delays (i.e. 5, 20, 60 s), a two-way repeated-measures ANOVA revealed no significant differences between species (*F*_(4,35)_ = 1.47 *p* = .252) or phylogenetic group (*F*_(3,37)_ = 0.93, *p* = .446), no significant differences between performances in the different time delays (*F*_(2,35)_ = 1.34, *p* = .274), and no interaction between species and time (*F*_(8,35)_ = 0.43, *p* = .898). In general, however, the subjects’ performances slightly declined with increasing time delays (see Table 3; Fig. 2).

Comparing the simultaneous and successive conditions, a two-way repeated-measures ANOVA revealed a significant interaction between species and condition (with species as between-subject factor and mean performances in the simultaneous and successive condition as dependent variables: *F*_(4,18)_ = 9.06, *p* < .001). Post hoc tests (Holm–Sidak method) showed no significant differences between the simultaneous and successive conditions for the bonobos (*p* = .554), chimpanzees (*p* = .165), and macaques (*p* = .722), but the baboons performed significantly better in the successive than in the simultaneous condition (*p* < .001). In contrast, the human subjects discriminated significantly smaller size differences in the simultaneous than in the successive conditions (*p* = .002).

### Discussion

Except for the gorillas, subjects of all species learned to choose the larger of two cubes. Furthermore, we did not find significant differences in the minimal sizes the successful species were able to discriminate, neither in the simultaneous nor in the successive condition. The better performance of the baboons in the second, successive condition was probably due to their familiarization with the general setup and a better understanding of the test situation. In fact, being able to choose the larger cube in the successive condition implies that they were also able to discriminate the cubes in the simultaneous condition. Indeed, in the motivational trials of the successive condition, when the cubes were presented simultaneously, all baboons chose the larger cube. In sum, the gorillas were outperformed by the other species regarding these fine-grained size discrimination abilities, but there were no significant differences between the other apes, monkeys, and humans. These results question the assumption of clear-cut differences between the phylogenetic groups and rather suggest differences within the great apes (see also Schmitt et al. 2012 for similar findings) To test whether gorillas have difficulties discriminating between two different sized objects in general, we conducted an additional experiment with larger size differences between the stimuli.

## Experiment 2: large size discrimination

In this experiment, we examined the abilities of gorillas, chimpanzees, bonobos, and macaques to discriminate two objects with larger differences in size (about 60 %). Furthermore, we included a control condition to exclude that the subjects took any hint from the experimenter or baiting procedure to solve the task. (As the group of baboons was transferred to another facility, we could not test them in this condition, but they had successfully discriminated 60 % size differences in Experiment 1).

### Methods

#### Subjects

Eight chimpanzees (3 males, 5 females), five bonobos (3 males, 2 female), six gorillas (2 males, 4 females), and seven long-tailed macaques (4 males and 3 females) participated in the study. All apes were housed at the Wolfgang Köhler Primate Research Center in Leipzig Zoo, the macaques were housed at the German Primate Center in Göttingen (s. Experiment 1 & Table S1).

#### Materials

The apparatus was the same as described above. Instead of the cubes, two different pairs of opaque containers were placed on the platform. An occluder was used to hide the baiting from the monkeys. We used two sets of containers:

*Size*: Two white plastic plant pots identical in shape but differing in size (Stimulus-Set 1: 90 mm high × 110 mm in diameter versus 120 mm high × 140 mm in diameter; Stimulus-Set 2: 100 mm high × 120 mm in diameter versus 140 mm high × 160 mm in diameter). The larger pots were approximately 60 % larger in volume than the smaller pots
*Control*: Two green or two orange plastic cups of identical size (90 mm high and 70 mm in diameter) and shape

#### Procedure

The experimenter placed the occluder, baited the larger pot with a reward, and placed the pots to the left and right side on the table. The occluder was lifted, and the subject was allowed to choose. If it chose the larger pot, it received the reward; if it chose the smaller one, it received nothing but was shown the place of the reward. The position of the baited object was pseudo-randomized with the restriction that the reward should not appear on the same side for more than two consecutive trials, but equally often left and right.

All subjects participated in a total of 96 trials in eight 12-trial sessions; four control trials were randomly interspersed within each session. Starting condition (i.e. Set 1 or Set 2) was randomised and balanced across individuals. Each individual received one or two sessions per day.

#### Data analyses

First, we calculated the mean percent of correct trials for each subject in the experimental and control conditions. To compare the performance of the different species, we conducted a mixed-design ANOVA with Stimulus-Set as random, within-subject variable, and species as fixed, between-subject variable. To compare the species’ performances in the experimental and control conditions to chance, we conducted paired *t*-tests. The alpha-level was set to 0.05.

### Results and discussion

**Fig. 3:**
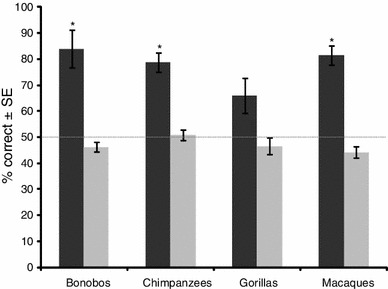
Percent of correct responses of the apes and monkeys in the test condition (*black bars*) and the control condition (*gray bars*) of Experiment 2. The *dotted line* represents the chance level. *p < .05

The mixed-design ANOVA revealed no significant differences between the species tested (*F*_(3,20)_ = 2.729, *p* = .217). Bonobos, chimpanzees, and macaques chose the larger pot significantly above chance (paired *t*-tests: bonobos *p* = .009; chimpanzees & macaques *p* < .001), while the gorillas’ choice revealed a trend toward choosing the larger pot (*p* = .063) (see Fig. 3). In the control condition, none of the subjects performed above chance, making it unlikely that they took any hint from the baiting procedure or the experimenter to solve the task (paired *t*-tests: bonobos *p* = .099; gorillas *p* = .319; chimpanzees *p* = .712). The macaques chose the correct cup even less often than expected by chance (paired* t*-test: *p* = .039).

## General discussion

In line with recent studies (e.g. Amici et al. 2010; Schmitt et al. 2012), we did not find clear-cut differences between the performances of humans, apes, and monkeys. Chimpanzees, bonobos, baboons, macaques, and humans performed on the same level when the cubes were presented simultaneously. They were able to recognize differences in volume of on average about 20 %, and three subjects even discriminated between 5 % size differences. As the cubes used were relatively small, this means discriminating even 1 mm differences in side length. In contrast to the remarkable similarities between the phylogenetic groups, we found differences within the great apes. Gorillas performed significantly worse in discriminating small size (volume) differences than all other species tested, thus also worse than the monkeys. In the control experiment (Exp. 2), when the size difference was large, the gorillas were much better in discriminating between the stimuli and performed only slightly worse than the other species, demonstrating that they probably do not have lower discriminatory abilities in general, but only problems discriminating between subtle size differences as in Experiment 1 (for a review on primate cognition see Tomasello and Call 1997).

Furthermore, we did not find any species differences in the successive conditions (gorillas were not tested, as they did not pass the simultaneous condition), when the cubes were only shown one after the other. All species were able to choose the larger cube even when they had never seen them simultaneously, suggesting that memorizing specific object features such as size has deep evolutionary roots. Notably, the tests with the human subjects revealed that their performance declined significantly when the cubes were presented in succession, highlighting the outstanding performance of the nonhuman subjects.

Accordingly, our results suggest that both monkeys and apes were able to form mental representations of the cubes, as they were able to pick the larger cube even after a 60 s delay, suggesting that they compared the objects internally. Whether monkeys are able to form mental representations is a controversial issue, as this capacity is often only assigned to apes (Byrne 2000). However, a recent quantity discrimination study in baboons and macaques also suggested that the monkeys’ performance was indeed influenced by their internal representations of the demonstrated stimuli and not by other physical properties (Schmitt and Fischer 2011). Additionally, Basile and Hampton (2011) recently demonstrated that rhesus macaques (*Macaca mulatta*) were able to reconstruct simple object shapes from memory. Thus, a basic capacity to mentally represent objects seems to be common in at least Old World primates.

The results further indicate that variation in brain size does not clearly correlate with such a basic ability as size discrimination. Baboons and macaques, having relatively smaller brains than bonobos and chimpanzees (Jerison 1973), performed just as well as the apes. The fact that the baboons had to be trained with a 100 % size difference at the beginning is likely due to their lack of experience. None of the animals had ever participated in an experiment before and they had to get accustomed to the testing situation itself to understand the task. Interestingly, however, gorillas have smaller relative brain sizes than the other species tested (Montgomery et al. 2011) and performed worst in the experiments. It may thus be that larger relative brain sizes can enhance the ability to discriminate differently sized objects at this small scale. Nonetheless, as we found no significant differences between the other species tested despite their large differences in brain size, additional factors may have influenced the performance of the gorillas, which we discuss below. Regarding the similar performances of the monkeys and apes, our findings corroborate a recent study, which also found no clear-cut differences between the phylogenetic groups in a variety of cognitive tasks (Schmitt et al. 2012). These studies thus challenge the view of a deep cognitive split between monkeys and apes (see also Amici et al. 2010; Tomasello and Call 1997) and suggest that differential socio-ecological pressures may have caused species differences (see also Amici et al. 2008).

In particular, females of some Old World primate species exhibit exaggerated sexual swellings during their fertile phase, and their fluctuating size should encode information on probability of ovulation, which in turn influences male sexual behavior and male–male competition for matings (Zinner et al. 2002, 2004). In such species, there may be a premium on (males’) ability to discriminate between swellings of different size. Our experiments showed that gorillas—not showing large swellings—were outperformed by the species exhibiting large sexual swellings (i.e. chimpanzees, bonobos, baboons, macaques). Both sexes of these species were able to detect size differences of 20 % and less, which corresponds to the observed changes in the period of maximum swellings in female chimpanzees (Deschner et al. 2004). However, humans performed on the same discriminatory level although they do not exhibit sexual swellings, suggesting that presence and size of sexual swellings did probably not significantly influence the evolution of species discriminatory abilities. Nonetheless, the size discrimination abilities observed in all species, but the gorillas can be put to use in the context of sexual selection, where males need to be able to discriminate between females based on different swelling sizes. However, our data do not indicate whether males would be able to discriminate sexual swellings by size if the time lag between two swellings exceeds the time lag used in the protocols of the current study, for example, hours or days.

The fact that the gorillas were outperformed by all other species may have been due to a number of additional factors, like for example, a lack of motivation to perform the task, as it is sometimes rather difficult to keep gorillas motivated in such kind of experiments. However, the gorillas took part in all 12 sessions of the initial experiment and did not stop participating, demonstrating their general interest in this experiment. Furthermore, as none of the gorillas passed the first size discrimination experiment irrespective of their experience with experimental testing, a lack of experience seems not to account for their failure. One additional and interesting aspect is, however, that gorillas were the least frugivorous species tested, often eating lots of leaves and foliage (Robbins 2011). It may be that a frugivorous diet may have promoted the evolution of size discrimination abilities. Being able to choose the larger of two fruit items can have a substantial influence on an animal’s fitness and evolution may have favoured individuals which could pick the larger fruit item when competing with conspecifics. The fact that color vision probably also evolved in response to frugivory demonstrates that diet can have an influence on species’ perceptual abilities (Osorio and Vorobyev 1996; see also Sussman et al. 2012). However, further tests with folivorous species, especially those that are truly folivorous such as howler monkeys, should be conducted to better understand the possible influence of feeding ecology on such basic discriminatory skills. Furthermore, gorillas were the largest species tested and it may be that the visual angle of the stimuli was slightly different for them compared to the other apes. As Troscianko et al. (2012) recently showed, morphological features such as binocular vision may strongly influence a species’ foraging behavior. Although we do not think that the rather small body differences between the great apes accounted for their different performance, testing them on, for example, a haptic version of the task in which the subjects have to discriminate between differently sized objects via touching would be an interesting comparison (see Hille et al. 2001; Kahrimanovic et al. 2011 for studies with monkeys and humans). Accordingly, various factors may have influenced species’ size discrimination abilities, as well as their motivation to rely on such cues in a specific situation. Although we could not disentangle these factors in our study, considering environmental and socio-ecological factors in comparative studies is essential as these probably influenced the evolution of perceptual and also cognitive capacities (Amici et al. 2008).

Taken together, our study shows that primates are able to notice and remember subtle differences between two objects, even after successive presentation. We found no differences between humans, apes, and monkeys, highlighting the fact that differences in cognitive abilities do not always map neatly onto phylogenetic relationships. In contrast, other environmental factors, such as diet, may better explain species differences. These findings emphasize the importance of conducting direct comparative analyses and cast doubt on the assumption that larger brains per se confer an advantage in such kinds of tests.

## Electronic supplementary material

Below is the link to the electronic supplementary material.

Supplementary material 1 (PDF 23 kb)
