# Supplementary material for: Monkeys perform as well as apes and humans in a size discrimination task
Source: Anim Cogn. 2013 Feb 27;16(5):829–38. doi: 10.1007/s10071-013-0616-0 (PMC3748326; doi:10.1007/s10071-013-0616-0)
Supplement: Supplementary file 1 — Supplementary material 1 (PDF 23 kb) [file 10071_2013_616_MOESM1_ESM.pdf]

Online Resource 1

Supplementary Table S1

Table S1: Name, gender, age and study participation of the nonhuman primates

Subject	Gender	Age (years)	Study participation
**Chimpanzee**			
Robert	M	30	2
Frodo	M	12	1,2
Patrick	M	9	1,2
Riet	F	28	2
Dorien	F	25	1,2
Sandra	F	13	2
Jahaga	F	13	2
Fifi	F	13	2
Natascha	F	unknown	1
Fraukje	F	unknown	1
**Bonobo**			
Joey	M	23	1,2
Limbuko	M	10	1,2
Kuno	M	8	1,2
Ulindi	F	12	1,2
Yasa	F	9	1
**Gorilla**			
Gorgo	M	25	1,2
Nkwango	M	9	2
Ndiki	F	28	1,2
Bebe	F	25	1,2
Viringika	F	11	1,2
Bianka	F	unknown	1
Hakuna	F	unknown	1
Lena	F	unknown	1
Ruby	F	unknown	1,2
**Baboon**			
Meister	M	7	1
Jago	M	2	1
Pünktchen	M	2	1
Tröpfchen	F	11	1
Nase	F	7	1
Schecki	F	6	1
Brille	F	5	1
Beinhaar	F	3	1
Tiger	F	3	1
**Macaque**			
Popey	M	4	2
Samson	M	3	1,2
Pit	M	3	1,2
Lenny	M	2	1,2
Sunny	F	9	1
Maja	F	4	1
Sally	F	4	1,2
Selina	F	3	2
Linda	F	2	1
Sophie	F	2	1,2
